# Acute Myeloid Leukemia with Occult Systemic Mastocytosis or Atypical Mast Cells Post-induction

**DOI:** 10.7759/cureus.3734

**Published:** 2018-12-14

**Authors:** Salahuddin Khan, Zain Abid, Humza F Siddiqui, Saima Zahoor, Ghulam Haider

**Affiliations:** 1 Oncology, Jinnah Postgraduate Medical Center, Karachi, PAK; 2 Oncology, Jinnah Postgraduate Medical Centre, Karachi, PAK

**Keywords:** systemic mastocytosis, acute myeloid leukemia, associated clonal hematologic non mast cell lineage (ahnmd)

## Abstract

Systemic mastocytosis (SM) is a state of disease that is related to the clonal, neoplastic proliferation of mast cells. Patients who present with SM-Acute Myeloid Leukemia (AML) often have the worst outcome. We present a case of an 18-year-old female who was diagnosed with AML (FLT3 (Fms like tyrosine kinase 3) and PML-RARA (promyelocytic leukemia-retinoic acid receptor alpha) translocation-negative) and after initial treatment with a standard induction regimen of cytarabine and daunorubicin (3+7 regimen), her bone marrow showed blast cells less than 5% and dense aggregates/sheets of atypical/immature mast cells with immunohistochemical stain CD117+ve and toluidine blue positive in mast cell aggregates. Mastocytosis is a clonal neoplastic proliferation of mast cells that accumulate in one or more organ system. Therefore, it is essential to diagnose systemic mastocytosis, particularly in patients of hematological neoplasms.

## Introduction

Systemic mastocytosis (SM) is an abnormal growth and accumulation of mast cells in extracutaneous organs, bone marrow being the most common site, with bone marrow biopsy and aspiration used in making the diagnosis [1]. Approximately 40% of patients with SM present with an associated clonal hematological non-mast cell lineage disorder [2-3]. Systemic mastocytosis with an associated, clonal, hematologic non-mast cell lineage disorder (SM-AHNMD) is the second most common type of mastocytosis [4].

a.       These non-mast cell lineage disorders may include myelodysplastic syndrome (MDS), myeloproliferative neoplasm (MPN), acute myeloid leukemia (AML), chronic myelogenous leukemia, plasma cell myeloma, non-Hodgkin lymphoma, and unclassifiable myelogenous malignancies [5].

b.       An associated AML has the worst prognosis [6].

We present a case of AML (M3), which after treatment showed atypical/immature mast cells in the post-induction marrow biopsy. The clinical course and prognosis of SM are variable and can range from plodding to aggressive.

## Case presentation

**Figure 1:**
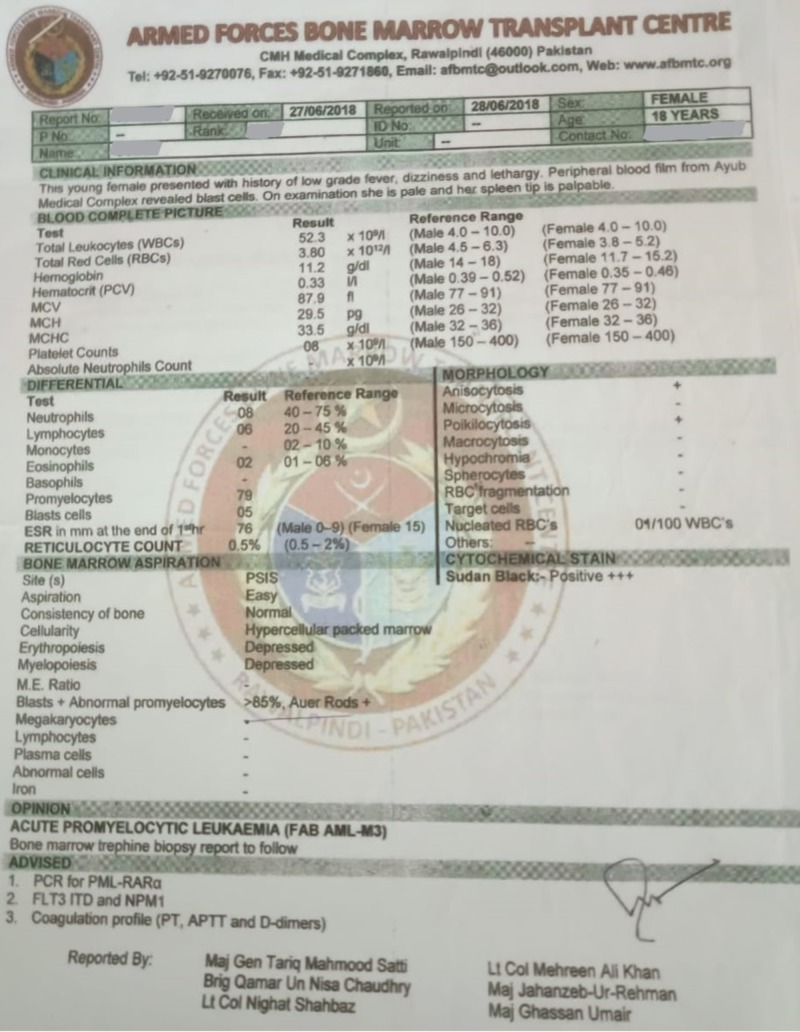
Bone marrow aspiration (pre-induction chemotherapy)

An 18-year-old female, married, presented with a history of low-grade fever, dizziness, and lethargy for three weeks. On examination, she was pale and her spleen was palpable. Her complete blood count revealed hemoglobin (Hb) of 11.2 gm/dI, total leukocyte count (TLC) of 52,300/mm^3^, and platelet count of 8000/mm^3^; her differential leukocyte count showed promyelocytes 79%. In Figure 1, the bone marrow aspiration showed blasts and abnormal promyelocytes 85%, and Auer rods were positive. The histochemical stain was positive for Sudan black, whereas erythropoiesis and myelopoiesis were depressed. The bone marrow trephine biopsy revealed sheets of blasts and abnormal promyelocytes consistent with acute promyelocytic leukemia (FAB AML M3 (French-American-British acute myeloid leukemia type 3)).

Molecular genetics, including the polymerase chain reaction (PCR) for AML gene markers and PCR for FLT3 ITD (Fms like tyrosine kinase 3-internal tandem duplication) mutations, were negative. The TP53 gene mutation by fluorescence in situ hybridization (FISH) was negative. Immunophenotyping for acute leukemia was done, but the report was insufficient in its description. Cytogenetics showed that a culture of lymphocytes at 37^o^C failed to yield any growth. Promyelocytic leukemia-retinoic acid receptor alpha (PML-RARA) translocation by PCR was done, which was not detected.

We gave induction chemotherapy (3+7 regimen). On Day 16, her complete blood count (CBC) showed improvement with hemoglobin (Hb) 11.4 gm/dI, white blood cell (WBC) 5,100/mm^3^, and platelet count 240,000/mm^3^. The bone trephine procedure was done on post-induction Day 28, in which the bone marrow aspirate showed tri-lineage hematopoiesis, with a prominent increase in erythroid precursors and an increase in mast cells that constituted around 7% of the total nucleated non-erythroid population. These mast cells were spindle-shaped with centric to eccentric nuclei with clumped chromatin and absent nucleoli. They were also present in aggregates with erythroid precursors and prominent in the spicules. Blast cells were less than 5%. Bone trephine (hematoxylin and eosin (H&E)) section showed overall cellularity of around 55% to 60%. Cellular areas exhibited tri-lineage hematopoiesis alternating with areas of dense aggregates/sheets of atypical/immature mast cells. Immunohistochemical stains of mast cell aggregates were CD117 positive. Toluidine blue in the mast cell aggregates was metachromatic positive. Overall, the post-induction bone marrow (BM)/trephine biopsy showed a clearance of blast cells and increased mastocytes, which was suggestive of systemic mastocytosis with an associated hematologic neoplasm (SM-AHN, systemic mastocytosis with associated acute myeloid leukemia (SM-AML)) according to the World Health Organization (WHO) 2016 classification of hematolymphoid neoplasms.

But clinically, according to updated WHO diagnostic criteria for systemic mastocytosis 2017, we cannot confirm the diagnosis of SM-AHN, as the mast cells were 7% in our patient while the criteria are >25%, and investigations like KIT-D816V or other activating point mutations of KIT and serum tryptase levels were also not available in our country. We started consolidation chemotherapy of the patient with high-dose cytarabine.

## Discussion

The diagnosis of SM-AHNMD may be difficult to establish because the associated malignancy may mask the histological and cytological features of system mastocytosis (SM). The diagnosis can only be made when there is clear morphologic evidence of both SM with multifocal tissue infiltrates and AHNMD [7]. Malignant mast cells may abnormally express CD2 and/or CD25, which may be detected by immunochemistry or flow cytometry, which was not done in our case because of an affordability issue. Activating c-kit mutations are considered the hallmark of neoplastic mast cells [8], which was not available in our country. In SM-AHNMD, AHNMD may be diagnosed before, simultaneously with/after the diagnosis of SM. In some cases, the diagnosis of SM is missed/masked at the time of diagnosis, mainly due to the excess number of blasts and the tendency of mast cells to localize within the stroma of bone marrow particles [9]. However, the persistence of mastocytosis at the time of post-induction BM evaluation with a reduction in blast percentage helps us to diagnose SM at that time [10-14].

## Conclusions

The diagnosis of SM is difficult to establish, as the associated malignancy may obscure the morphological features of SM. It is essential to diagnose SM, particularly in AHNMD, which may be difficult to diagnose during the course of the disease. However, a reduction in blast cell percentage at the time of a post-induction marrow evaluation helps in diagnosis.
